# Bulk and interfacial properties of decane in the presence of carbon dioxide, methane, and their mixture

**DOI:** 10.1038/s41598-019-56378-y

**Published:** 2019-12-24

**Authors:** Nilesh Choudhary, Arun Kumar Narayanan Nair, Mohd Fuad Anwari Che Ruslan, Shuyu Sun

**Affiliations:** Physical Science and Engineering Division (PSE), Computational Transport Phenomena Laboratory, King Abdullah University of Science and Technology (KAUST), Thuwal, 23955-6900 Saudi Arabia

**Keywords:** Climate-change mitigation, Thermodynamics, Surfaces, interfaces and thin films

## Abstract

Molecular dynamics simulations were performed to study the bulk and interfacial properties of methane + n-decane, carbon dioxide + n-decane, and methane + carbon dioxide + n-decane systems under geological conditions. In addition, theoretical calculations using the predictive Peng-Robinson equation of state and density gradient theory are carried out to compare with the simulation data. A key finding is the preferential dissolution in the decane-rich phase and adsorption at the interface for carbon dioxide from the methane/carbon dioxide mixture. In general, both the gas solubility and the swelling factor increase with increasing pressure and decreasing temperature. Interestingly, the methane solubility and the swelling of the methane + n-decane system are not strongly influenced by temperature. Our results also show that the presence of methane increases the interfacial tension (IFT) of the carbon dioxide + n-decane system. Typically, the IFT of the studied systems decreases with increasing pressure and temperature. The relatively higher surface excess of the carbon dioxide + n-decane system results in a steeper decrease in its IFT as a function of pressure. Such systematic investigations may help to understand the behavior of the carbon dioxide-oil system in the presence of impurities such as methane for the design and operation of carbon capture and storage and enhanced oil recovery processes.

## Introduction

Since the beginning of the industrial revolution in the mid-1700s, driven by the burning of fossil fuels, there has been a steady increase in the emission of the greenhouse gas CO_2_ into the atmosphere. According to recent reports, anthropogenic CO_2_ emissions are more than 30 Gt per year, primarily from the combustion of fossil fuels^1^. Anthropogenic CO_2_ emissions play an important role in global warming and the continued increase in the amount of CO_2_ in the atmosphere is predicted to lead to significant environmental issues^2–5^. For example, oceans are becoming warmer and more acidic, and sea levels are rising rapidly as global warming melts glaciers and ice sheets. The Intergovernmental Panel on Climate Change projects a sea-level rise of 28–61 cm by 2100, if greenhouse gas emissions are significantly reduced^4^. At the Paris climate conference^6^, nearly 200 countries agreed to a target of keeping global warming below 2 °C above the pre-industrial average. In this context, carbon capture and storage (CCS) is considered to be a key technology for reducing anthropogenic CO_2_ emissions. A variety of materials have been considered for CO_2_ capture including metal-organic frameworks^7–9^, zeolites^8,10^, zeolitic imidazolate frameworks^11^, polymers^10,12–14^, and geological formations^15–26^. CCS will complement other crucial technologies, such as the use of renewable energy (solar, wind, etc.), increasing energy efficiency, and switching to low-carbon fuels.

Since many decades, enhanced oil recovery (EOR) techniques have been used in order to improve both oil field production and CO_2_ sequestration^19–26^. In 2013, CO_2_-EOR provided about 4% (0.28 million barrels per day) of the total U.S. crude oil production and its contribution is projected to increase in the future^26^. Properties such as interfacial tension (IFT), density, miscibility, and solubility of the CO_2_-oil system are key for CO_2_-EOR. For example, a decrease in the IFT generally resulted in an increase in the oil yield of the CO_2_ flooding process. Typically, the miscible CO_2_ flooding process recovers 10–20% of the original oil in place (OOIP), whereas the immiscible CO_2_ flooding process recovers 5–10% of the OOIP due to the interfacial tension between CO_2_ and oil^24^. Traditionally, natural sources of CO_2_ are used for about 90% of EOR production in the U.S^23^. However, EOR using anthropogenic CO_2_ emissions would be necessary to achieve the desired climate benefits. CO_2_ captured from power plants and industrial sources may contain impurities^27–29^. The cost of purification of gas mixtures considerably increases for obtaining a product of high degree of purity. Therefore, it is important to the study the behavior of the CO_2_-oil system in the presence of impurities such as methane for the design and operation of CCS and EOR processes.

The bulk and interfacial properties of binary mixtures of methane or carbon dioxide with alkanes such as n-decane (model oil in this work) under reservoir conditions receive continuous attention and many experimental^30–42^, theoretical^38–41,43–51^, and simulation^39,46,52–56^ studies have been reported. These studies, for example, showed that the mole fractions of methane and carbon dioxide in the decane-rich phase increase with pressure. In general, the IFT of CH_4_ + n-decane and CO_2_ + n-decane systems decreases with increasing pressure and temperature. However, at high pressures, the IFT of the CO_2_ + n-decane system increased with increasing temperature^37,40,41,50^. This can be explained by the fact that the local adsorption of gas molecules at the interface is more pronounced for carbon dioxide when compared to that of methane, especially at low temperatures. It is to be noted that, the investigation of ternary mixtures containing methane, carbon dioxide, and higher alkanes has been rare^57^.

Molecular simulations provide valuable insights into the bulk and interfacial properties of various systems^14–17,58–63^. In this work, we perform molecular dynamics (MD) simulations to understand the properties of CH_4_ + n-decane, CO_2_ + n-decane, and CH_4_ + CO_2_ + n-decane systems over a broad range of temperature (313–442 K) and pressure (up to about 300 bar) relevant to geological processes. Given the general lack of experimental results for these systems, especially the ternary case, theoretical modeling is employed to complement the simulation data.

## Methods

### Simulation details

**Figure 1:**
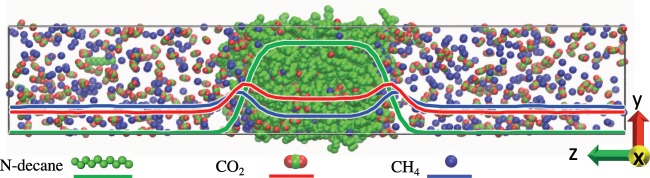
Equilibrium snapshot of the CH_4_ + CO_2_ + n-decane system at 343 K and 60 bar.

The MD simulation was carried out using the GROMACS code^64^. The simulation model and method used here are similar to the ones reported in our previous work^14,63^. The transferable potentials for phase equilibria force field^65^ was used to model n-decane, methane, and CO_2_. The simulations were initiated by placing 400 decane molecules and up to 2048 CH_4_/CO_2_ molecules in each simulation box. In directions parallel to the interface, each cell was about 50 × 50 Å, and periodic boundary conditions were used in all directions (Fig. 1). These values were chosen to ensure reasonable bulk phases with enough molecules and also to reduce the truncation and system size effects in the calculations of the bulk and interfacial properties^39,46,52–56^. These systems are energy-minimized using the steepest descent algorithm and subjected to equilibration MD runs of 10 ns in the *NPT* ensemble. During the *NPT* runs, the volume changes are obtained by adjusting only the cell size in the *z* direction (*L*_*z*_) normal to the interface. After that we carried out production runs of 6 ns duration in the *NVE* ensemble. The leap-frog algorithm^66^ with a time step of 1 fs was used to integrate the equations of motion. The linear constraint solver algorithm^67^ was implemented to constrain the bond lengths and angles. Virtual sites were used to maintain the rigid structure of CO_2_^68^. The particle mesh Ewald method was used to calculate the long-range electrostatic interactions. The Nosé-Hoover thermostat and Parrinello-Rahman barostat, with a relaxation time of 2.0 ps for both, were used to regulate the temperature and pressure, respectively. Each MD run was repeated three times and the average value was taken.

The IFT was calculated as follows^52,63^:

1 $$\gamma =\frac{1}{2}{L}_{z}[{P}_{zz}-\frac{1}{2}({P}_{xx}+{P}_{yy})],$$

where *P*_*xx*_, *P*_*yy*_, and *P*_*zz*_ are the three diagonal components of the pressure tensor. Typically, for the estimation of various atomic density profiles, a bin width of 1 Å is used.

### Theoretical details

The volume translated predictive Peng-Robinson 1978 (VT-PPR78)^40,44,69^ equation of state (EoS) with the usual van der Waals one-fluid mixing rules and a linear mixing rule for the volume correction is used to estimate bulk properties. The VT-PPR78 model calculates the binary interaction parameter *k*_*ij*_ for each temperature using a group contribution method. Additionally, the interfacial properties were calculated by coupling the VT-PPR78 EoS with the density gradient theory (DGT). In brief, the Helmholtz free energy of the planar interface is given by^70,71^

2 $$F=A{\int }_{-\infty }^{+\infty }[{f}_{0}(n)+\frac{1}{2}\sum _{i}\sum _{j}\,{c}_{ij}\frac{d{n}_{i}}{dz}\frac{d{n}_{j}}{dz}]dz,$$

where *A* is the area of the planar interface, *f*_0_ represents the Helmholtz free energy density of the homogeneous fluid at the local density *n*, *dn*_*i*_/*dz* is the local gradient in density of a given component *i*, and *c*_*ij*_ is the cross influence parameter defined as follows:

3 $${c}_{ij}=(1-{\beta }_{ij})\sqrt{{c}_{ii}{c}_{jj}},$$

where *c*_*ii*_ and *c*_*jj*_ denote the pure component influence parameters, and *β*_*ij*_ represents the binary interaction coefficient. In this study, the pure component influence parameter is computed based on the correlation developed by Miqueu *et al*.^44^. The minimization of the Helmholtz free energy leads to the following Euler-Lagrange equations:

4 $$\sum _{j}\,{c}_{ij}\frac{{d}^{2}{n}_{j}}{d{z}^{2}}={\mu }_{i}^{0}({n}_{1}(z),\ldots ,{n}_{{N}_{c}}(z))-{\mu }_{i}\,{\rm{for}}\,i,j=1,\mathrm{.}.,{N}_{c},$$

where ${\mu }_{i}^{0}\equiv {(\frac{\partial {f}_{0}}{\partial {n}_{i}})}_{T,V,{n}_{j}}$, *μ*_*i*_ denotes the chemical potential of component *i* in the equilibrium bulk phase and *N*_*c*_ represents the total number of components. To obtain the density profiles across the interface, the above set of equations must be solved together with the following boundary conditions:

5 $$\begin{array}{l}{n}_{i}(\,-\,\infty )={n}_{i}^{I}\\ {n}_{i}(\,+\,\infty )={n}_{i}^{II}\end{array}$$

where ${n}_{i}^{I}$ and ${n}_{i}^{II}$ are the equilibrium densities of component *i* in the coexisting bulk phases. As stated above, the bulk properties are calculted using the VT-PPR78 EoS. Using the geometric mixing rule (*β*_*ij*_ =0) reduces the system of *N*_*c*_ differential equations (Eq. (4)) to the system of (*N*_*c*_ − 1) algebraic equations:

6 $$\sqrt{{c}_{i}}({\mu }_{{\rm{ref}}}^{0}(n(z))-{\mu }_{{\rm{ref}}})=\sqrt{{c}_{ref}}({\mu }_{i}^{0}(n(z))-{\mu }_{i});i=1,\ldots ,{N}_{c},\,{\rm{and}}\,i\ne {\rm{ref}}.$$

These equations could compute the densities of all except one component and the density of the latter is monotonic function of *z* over the whole interface^40,44^ and used as a reference variable. Once the density profiles are known, the IFT can be calculated using the following equation:

7 $$\gamma ={\int }_{{n}_{{\rm{ref}}}^{I}}^{{n}_{{\rm{ref}}}^{II}}\,\sqrt{2\Delta \Omega (n)\sum _{i}\sum _{j}{c}_{ij}\frac{d{n}_{i}}{d{n}_{{\rm{ref}}}}\frac{d{n}_{j}}{d{n}_{{\rm{ref}}}}}d{n}_{{\rm{ref}}},$$

where

8 $$\Delta \Omega ={f}_{0}(n)-\sum _{i}\,{n}_{i}{\mu }_{i}+P.$$

Note that, ΔΩ(*n*) = Ω(*n*) − Ω_*B*_ is the difference between the grand thermodynamic potential $\Omega \equiv {f}_{0}(n)-\sum _{i}\,{n}_{i}{\mu }_{i}$ at the local density and its value in the bulk phase Ω_*B*_ = −*P*. Furthermore, the *z*-coordinate for the density profile can be computed using the following equation:

9 $$z={z}_{0}+{\int }_{{n}_{{\rm{ref}}}^{0}}^{{n}_{{\rm{ref}}}(z)}\,\sqrt{\frac{\sum _{i}\,\sum _{j}\,{c}_{ij}\frac{d{n}_{i}}{d{n}_{{\rm{ref}}}}\frac{d{n}_{j}}{d{n}_{{\rm{ref}}}}}{2\Delta \Omega (n)}}d{n}_{{\rm{ref}}},$$

where *z*_0_ and ${n}_{{\rm{ref}}}^{0}$ represent the coordinate and the density, respectively, arbitrarily chosen as origins.

## Results

### Atomic density profiles

**Figure 2:**
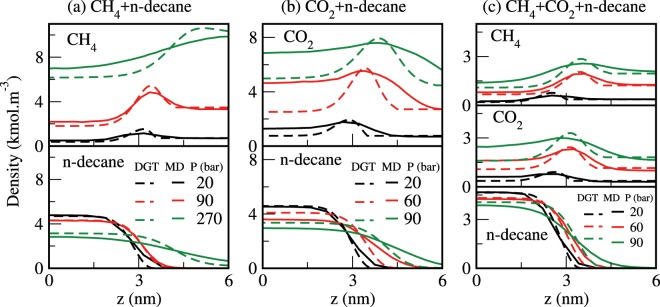
Atomic density profiles for (**a**) CH_4_ + n-decane, (**b**) CO_2_ + n-decane, and (**c**) CH_4_ + CO_2_ + n-decane systems at 343 K. The solid lines represent the results from the MD simulations and the estimates obtained using the DGT with VT-PPR78 EoS are shown as dashed lines.

Both MD simulations and DFT calculations were used to predict the atomic density profiles that are not always easily accessible to experiments. For instance, Fig. 2 displays the density profiles of various species as obtained from the MD simulations (solid lines) and the corresponding theoretical data (dashed lines) for CH_4_ + n-decane, CO_2_ + n-decane, and CH_4_ + CO_2_ + n-decane (equimolar CH_4_/CO_2_ mixture) systems at 343 K. We see that the simulation results are in good qualitative agreement with the theoretical data. Note that among the different species, the maximum difference (differ by a factor of up to ~2) between the simulated and calculated densities is obtained for CO_2_, especially at high pressures. In all cases, the density profiles show local enrichment of gas molecules at the interface, but no such behavior is seen for n-decane in agreement with the previous studies^38–41,43–53^. That is, a positive surface activity is seen for both methane and carbon dioxide (*dn*_*i*_/*dz* = 0; *d*^2^*n*_*i*_/*dz*^2^ < 0 in the interfacial region). Whereas the density profile of decane across the interface varies monotonically and does not show surface activity. Also, the higher methane and/or carbon dioxide contents result in a broadening of the interface. As seen from the adsorption peaks in Fig. 2, the local accumulations of methane and carbon dioxide, in general, increase with pressure. However, at high pressures, the local enrichment of gas molecules decreases with pressure. It is known that the local enrichment of the interface in methane and carbon dioxide typically decreases with temperature^38–40,43–49,52,53^. The density of CO_2_ is usually higher in the liquid phase (decane-rich) when compared to that in the CO_2_-rich phase, whereas an opposite trend is found for methane. Our results also show the preferential dissolution in the decane-rich phase and adsorption at the interface for carbon dioxide from the CH_4_/CO_2_ mixture.

### Bulk properties

**Figure 3:**
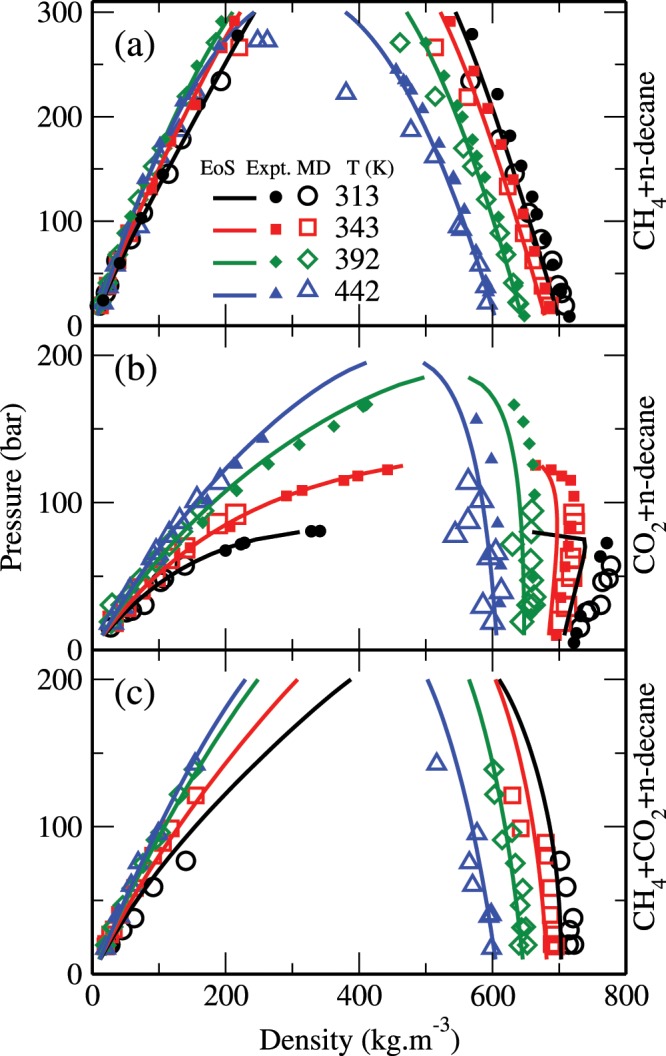
Saturated densities for (**a**) CH_4_ + n-decane, (**b**) CO_2_ + n-decane, and (**c**) CH_4_ + CO_2_ + n-decane systems. The open symbols represent the results from the MD simulations and the estimates obtained using the VT-PPR78 EoS are shown as lines. The experimental results of Pereira *et al*.^40^ are shown as solid symbols. The error bars are smaller than the symbol size.

Figure 3 shows the bulk densities as obtained from the MD simulations (open symbols) and the corresponding theoretical data (solid lines) for CH_4_ + n-decane, CO_2_ + n-decane, and CH_4_ + CO_2_ + n-decane (equimolar CH_4_/CO_2_ mixture) systems under geological conditions. These results are also compared with the corresponding experimental data^40^ (closed symbols). Other experimental data^30,32,33,35,39^ are similar to the ones plotted here and not shown for clarity. Note that in the absence of any experimental data, the simulation results of the CH_4_ + CO_2_ + n-decane system are only compared with the theoretical predictions. We see that both our theoretical and simulation results are in good agreement with the experimental data. For example, the overall absolute average deviation between theoretical and experimental density values is less than about 2% in the decane-rich phase, while it is less than about 3% in the CH_4_-rich or CO_2_-rich phase. In all cases, these total densities generally decrease with temperature in both phases. Also, these densities generally decrease with pressure in the decane-rich phase and increase with pressure in the CH_4_-rich and/or CO_2_-rich phases. However, for example, in the CO_2_ + n-decane system, the density of the decane-rich phase increases with pressure, especially at low temperatures and pressures. These different scenarios may take place depending on the gas solubility and swelling in the decane-rich phase.

**Figure 4:**
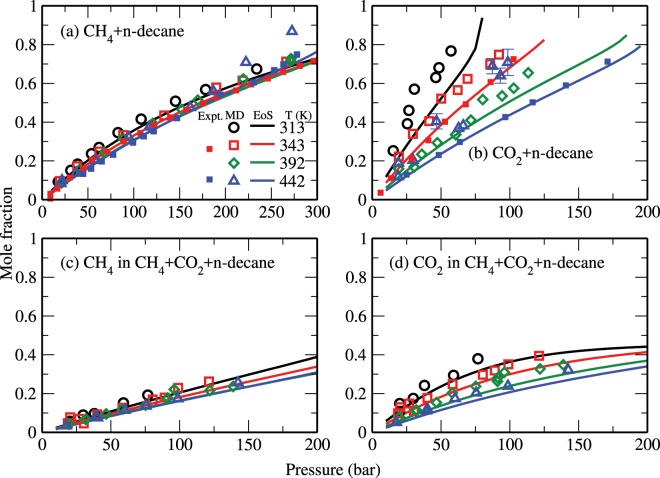
Mole fractions of CH_4_ and CO_2_ in the decane-rich phase: (**a**) CH_4_ in CH_4_ + n-decane, (**b**) CO_2_ in CO_2_ + n-decane, (**c**) CH_4_ in CH_4_ + CO_2_ + n-decane, and (**d**) CO_2_ in CH_4_ + CO_2_ + n-decane systems. The symbols are the same as in Fig. 3. Note that here the experimental data were taken from Reamer *et al*.^30,32^.

The knowledge of the species mole fractions and swelling effects plays an important role in the use of carbon dioxide for the recovery of hydrocarbon fluids from underground reservoirs^30,32,40,53,56^. Figure 4 shows the bulk mole fractions of methane and carbon dioxide in the decane-rich phase as obtained from the MD simulations (open symbols) and the corresponding theoretical data (solid lines) for all studied systems. These results are also compared with the experimental data^30,32^ (closed symbols). Both our theoretical and simulation results are again consistent with the experimental data. In general, various mole fractions for all studied systems increase with increasing pressure and decreasing temperature. Notably, the mole fraction of methane in these systems is not significantly affected by temperature. Overall, the amount of n-decane in the CH_4_-rich and/or CO_2_-rich phases was estimated to be relatively small (see, e.g., Fig. 2).

**Figure 5:**
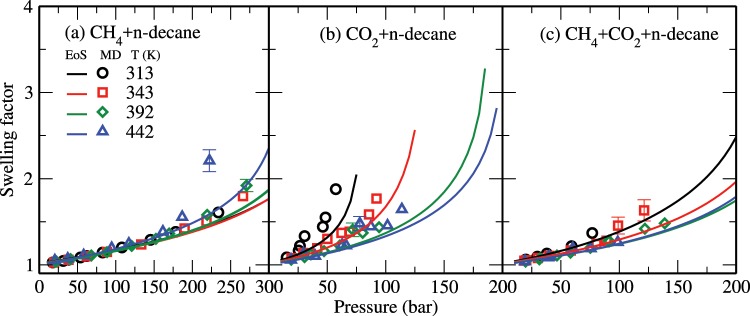
Swelling factor of (**a**) CH_4_ + n-decane, (**b**) CO_2_ + n-decane, and (**c**) CH_4_ + CO_2_ + n-decane systems. The symbols are the same as in Fig. 3.

**Figure 6:**
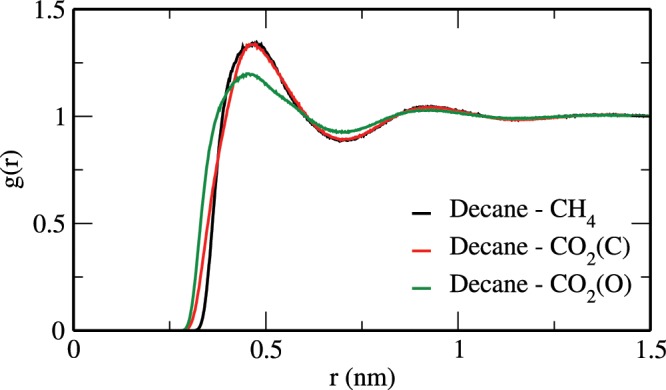
Radial distribution functions between decane and CH_4_/CO_2_ in the bulk liquid phase of the CH_4_ + CO_2_ + n-decane system at 343 K and 60 bar.

Furthermore, Fig. 5 shows the swelling factor for the decane-rich phase as obtained from the MD simulations (open symbols) and the corresponding theoretical data (solid lines) for all studied systems. The swelling factor is defined as ratio of the volume of the saturated decane-rich system to the volume of the decane alone^53,56^. Though not shown, both theoretical and simulation results for densities of the pure n-decane system at the studied conditions are in good quantitative agreement with the NIST data^72^. In general, the swelling factor for all the studied systems increases with increasing pressure and decreasing temperature, which is consistent with the above solubility data. Also, similar to the solubility vs temperature behavior, the swelling of the CH_4_ + n-decane system is not significantly affected by temperature. It is known that the interaction between CO_2_ and alkane molecules plays a key role in the solubility and swelling properties of the alkane-CO_2_ system^53,56^. For example, Fig. 6 shows the radial distribution functions between decane (carbon site) and CH_4_/CO_2_ in the bulk liquid phase of the CH_4_ + CO_2_ + n-decane system at 343 K and 60 bar. These results were obtained using an *NPT* MD simulation of the corresponding bulk liquid phase alone. From this figure, it can be seen that CO_2_ molecules are positioned relatively close to decane. Furthermore, for this system, we find that the interaction energies of decane-CO_2_ and decane-CH_4_ are about −2428.5 and −930.9 kJ/mol, respectively. All these results indicate the preferential interaction of decane with CO_2_ relative to CH_4_.

The conformation of the n-decane molecule may be characterized by its radius of gyration *R*_*g*_^54,55,61^. The *R*_*g*_ is defined as the root-mean-square distance of all atoms from their common center of mass. The *R*_*g*_ was calculated from the MD simulations in order to check the compactness of the conformations. Our calculations find that the *R*_*g*_ values decrease with increasing temperature and are in the range of 0.32–0.34 nm for all systems. This is because as the temperature increases, the effective monomer-monomer attraction becomes increasingly important, causing the chains to shrink^54^. The values of *R*_*g*_ are in good agreement with the previous reported results^54,55^. We also find that the *R*_*g*_ is much less sensitive to changes in pressure and to the presence of methane and CO_2_.

### Interfacial properties

**Figure 7:**
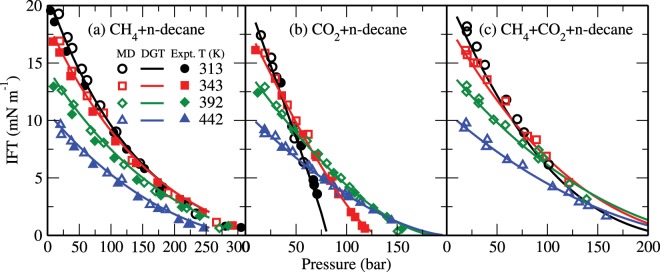
IFTs of (**a**) CH_4_ + n-decane, (**b**) CO_2_ + n-decane, and (**c**) CH_4_ + CO_2_ + n-decane systems. The open symbols represent the results from the MD simulations and the estimates obtained using the DGT with VT-PPR78 EoS are shown as lines. The experimental results of Pereira *et al*.^40^ are shown as solid symbols.

Figure 7 shows the IFTs as obtained from the MD simulations (open symbols) and the corresponding theoretical data (solid lines) for CH_4_ + n-decane, CO_2_ + n-decane, and CH_4_ + CO_2_ + n-decane (equimolar CH_4_/CO_2_ mixture) systems under geological conditions. These results are also compared with the corresponding experimental data^40^ (closed symbols). Other experimental data^31,33–35,37,39,41^ are similar to the ones plotted here and not shown for clarity. Note that in the absence of any experimental data, the simulation results of the CH_4_ + CO_2_ + n-decane system are only compared with the theoretical predictions. In the temperature range (313–442 K) considered here, the surface tension of pure n-decane decreases from about 22 to 11 mN/m^72^. It can be seen that both our theoretical and simulation results are in good agreement with the experimental estimates. For example, the overall absolute average deviation between theoretical and experimental IFT values is less than about 9%. These results show that, at a given temperature and pressure, the presence of CH_4_ increases the IFT of the CO_2_ + n-decane system. The IFT of CH_4_ + n-decane, CO_2_ + n-decane, and CH_4_ + CO_2_ + n-decane systems decreases with pressure. In general, a similar trend is observed for the temperature dependence of the IFT. However at high pressures, for example, the IFT of the CO_2_ + n-decane system increases with increasing temperature. This can be described^40^ by the fact that the local enrichment of gas molecules at the interface (see, e.g., Fig. 2) is more pronounced for carbon dioxide when compared to that of methane, especially at low temperatures. The correlation between the IFT and the local enrichment of the interface in methane and carbon dioxide will be discussed in detail below. Notably, theoretical calculations based on other equations of state^38–41,43–51^, such as the statistical associating fluid theory, can also give accurate estimates of the bulk and interfacial properties of these binary systems. One of the main properties governing the design and operation of CCS and EOR processes is the miscibility of the injected CO_2_ with oil^24,36^. The minimum miscibility pressure is taken as a pressure at which the IFT becomes zero^36^. Due to the fact that the IFT of the CO_2_ + n-decane system decreases steeply with increasing pressure, pure CO_2_ is completely miscible with decane from relatively low pressures. In the presence of methane, the CO_2_ + decane system seems to indicate a smaller miscibility region with respect to pressure.

## Discussion

The results obtained in this study allow one to quantify the the local enrichment of the interface in methane and carbon dioxide. In this context, it is important to notice that the IFT is related to the surface excess Γ_*i*_ as given by the Gibbs adsorption equation^40,63^:

10 $$-d\gamma =\sum _{i}\,{\Gamma }_{i}d{\mu }_{i}.$$

The surface excess of a component *i* with respect to decane is given by

11 $${\Gamma }_{i,{\rm{decane}}}=-\,{\alpha }_{i}{\int }_{-\infty }^{+\infty }\Delta C(z)dz,$$

with

12 $$\Delta C(z)=\frac{{n}_{{\rm{decane}}}(z)-{n}_{{\rm{decane}}}^{II}}{{\alpha }_{{\rm{decane}}}}-\frac{{n}_{i}(z)-{n}_{i}^{II}}{{\alpha }_{i}},$$

where

13 $${\alpha }_{i}=\frac{{n}_{i}^{II}-{n}_{i}^{I}}{{n}_{i}^{II}-{n}_{i}^{I}+{n}_{{\rm{decane}}}^{II}-{n}_{{\rm{decane}}}^{I}},$$

and

14 $${\alpha }_{{\rm{decane}}}=\frac{{n}_{{\rm{decane}}}^{II}-{n}_{{\rm{decane}}}^{I}}{{n}_{i}^{II}-{n}_{i}^{I}+{n}_{{\rm{decane}}}^{II}-{n}_{{\rm{decane}}}^{I}}.$$

**Figure 8:**
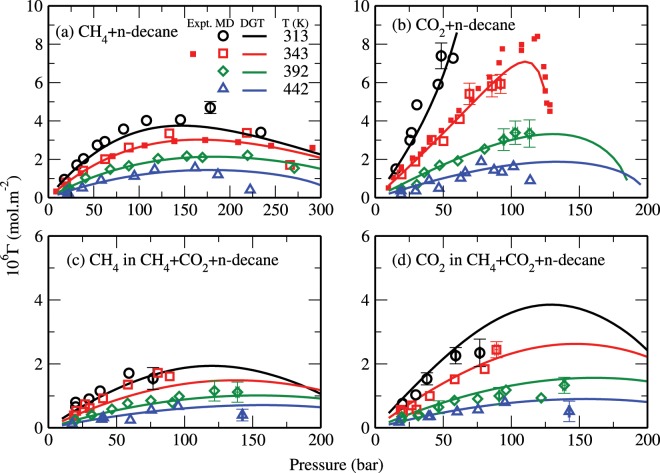
Surface excess of (**a**) CH_4_ in CH_4_ + n-decane, (**b**) CO_2_ in CO_2_ + n-decane, (**c**) CH_4_ in CH_4_ + CO_2_ + n-decane, and (**d**) CO_2_ in CH_4_ + CO_2_ + n-decane systems. The symbols are the same as in Fig. 7.

Here, *I* represents the CH_4_-rich and/or CO_2_-rich phases, and *II* the decane-rich phase. Figure 8 shows the surface excesses of CH_4_ and CO_2_ as obtained from the MD simulations (open symbols) and the corresponding theoretical data (solid lines) for all studied systems. These results are also compared with the experimental data^35,40^ (closed symbols). In all cases, we see a nonmonotonic variation of the surface excess with pressure. The relatively higher surface excess of the CO_2_ + n-decane system results in a steeper decrease in its IFT as a function of pressure (see also Eq. (10) and Fig. 7). Overall, the surface excesses decrease with increasing temperature and the results of the ternary system fall between those of the binary systems.

To conclude, we have studied using MD simulations the bulk and interfacial properties of CH_4_ + n-decane, CO_2_ + n-decane, and CH_4_ + CO_2_ + n-decane systems under geological conditions. Theoretical modeling using the VT-PPR78 EoS combined with DGT was employed to complement the simulation data. The simulation results of the atomic density profiles and the IFT values are in good agreement with both the theoretical and experimental data. The results of our study show that, in general, the local accumulations of methane and carbon dioxide increase with increasing pressure and decreasing temperature. At high pressures, however, the local enrichment of gas molecules decreases with pressure. An important result is the preferential dissolution in the decane-rich phase and adsorption at the interface for carbon dioxide from the CH_4_/CO_2_ mixture. The gas solubility and swelling in the decane-rich phase can play a key role in determining the behavior of the bulk densities as a function of temperature and pressure. Typically, both the gas solubility and the swelling factor for all the studied systems increase with increasing pressure and decreasing temperature. Notably, the mole fraction of methane and the swelling of the CH_4_ + n-decane system are not significantly affected by temperature. Furthermore, we find that the presence of CH_4_ increases the IFT of the CO_2_ + n-decane system. The IFT of the investigated systems decreases with pressure. In general, a similar trend was observed for the temperature dependence of the IFT. However, at high pressures the IFT of, e.g., the CO_2_ + n-decane system increases with temperature. There is a nonmonotonic variation of the surface excess with pressure and it decreases with temperature. The surface excess is more pronounced for carbon dioxide when compared to that of methane in the studied systems. Because of this fact, the IFT decreases steeply with increasing pressure for systems containing CO_2_.
